# Different Spatial and Temporal Roles of Monocytes and Monocyte-Derived Cells in the Pathogenesis of an Imiquimod Induced Lupus Model

**DOI:** 10.3389/fimmu.2022.764557

**Published:** 2022-03-15

**Authors:** Atsushi Nomura, Miho Mizuno, Daisuke Noto, Aki Aoyama, Taiga Kuga, Goh Murayama, Asako Chiba, Sachiko Miyake

**Affiliations:** ^1^ Department of Immunology, Juntendo University Graduate School of Medicine, Tokyo, Japan; ^2^ Department of Internal Medicine and Rheumatology, Juntendo University School of Medicine, Tokyo, Japan

**Keywords:** classical monocytes, non-classical monocytes, imiquimod, toll-like receptor 7, resident macrophages, systemic lupus erythematosus, lupus nephritis

## Abstract

Mounting evidence indicates the importance of aberrant Toll-like receptor 7 (TLR7) signaling in the pathogenesis of systemic lupus erythematosus (SLE). However, the mechanism of disease progression remains unclear. An imiquimod (IMQ)-induced lupus model was used to analyze the lupus mechanism related to the aberrant TLR7 signals. C57BL/6 mice and NZB/NZW mice were treated with topical IMQ, and peripheral blood, draining lymph nodes, and kidneys were analyzed focusing on monocytes and monocyte-related cells. Monocytes expressed intermediate to high levels of TLR7, and the long-term application of IMQ increased Ly6C^lo^ monocytes in the peripheral blood and Ly6C^lo^ monocyte-like cells in the lymph nodes and kidneys, whereas Ly6C^hi^ monocyte-like cell numbers were increased in lymph nodes. Ly6C^lo^ monocyte-like cells in the kidneys of IMQ-induced lupus mice were supplied by bone marrow-derived cells as demonstrated using a bone marrow chimera. Ly6C^lo^ monocytes obtained from IMQ-induced lupus mice had upregulated adhesion molecule-related genes, and after adoptive transfer, they showed greater infiltration into the kidneys compared with controls. RNA-seq and *post hoc* PCR analyses revealed Ly6C^lo^ monocyte-like cells in the kidneys of IMQ-induced lupus mice had upregulated macrophage-related genes compared with peripheral blood Ly6C^lo^ monocytes and downregulated genes compared with kidney macrophages (MF). Ly6C^lo^ monocyte-like cells in the kidneys upregulated *Il6* and chemoattracting genes including *Ccl5* and *Cxcl13*. The higher expression of *Il6* in Ly6C^lo^ monocyte-like cells compared with MF suggested these cells were more inflammatory than MF. However, MF in IMQ-induced lupus mice were characterized by their high expression of *Cxcl13*. Genes of proinflammatory cytokines in Ly6C^hi^ and Ly6C^lo^ monocytes were upregulated by stimulation with IMQ but only Ly6C^hi^ monocytes upregulated IFN-α genes upon stimulation with 2′3′-cyclic-GMP-AMP, an agonist of stimulator of interferon genes. Ly6C^hi^ and Ly6C^lo^ monocytes in IMQ-induced lupus mice had different features. Ly6C^hi^ monocytes responded in the lymph nodes of locally stimulated sites and had a higher expression of IFN-α upon stimulation, whereas Ly6C^lo^ monocytes were induced slowly and tended to infiltrate into the kidneys. Infiltrated monocytes in the kidneys likely followed a trajectory through inflammatory monocyte-like cells to MF, which were then involved in the development of nephritis.

## Introduction

Systemic lupus erythematosus (SLE) is an autoimmune disease characterized by the production of many types of autoantibodies, especially anti-nuclear antibodies (1). Despite the postulated central role of B cells and autoantibodies in SLE, many interventions that target this compartment have not succeeded (2). Mounting evidence indicates the importance of aberrant innate immune signals in the pathogenesis of SLE (3), and symptoms such as neuropsychiatric disorders or nephritis are thought to be partly caused by innate immune mechanisms independent of acquired immunity (4, 5). Among the innate immune signaling molecules, polymorphisms of Toll-like receptor 7 (TLR7) were reported to have a significant association with the development of SLE (6), and the epicutaneous application of TLR7 induced lupus-like disease in mice (7). In that model, plasmacytoid dendritic cells (pDC) were accumulated in the TLR7 agonist-treated skin. pDC have critical roles in the pathogenesis of SLE through the production of IFN-α following TLR7 activation (8). Other than pDC, a study of SLE reported a link between TLR7 and immune cells including B cells (9), monocytes, and monocyte-derived cells (10, 11). Currently, a potential relationship between TLR7 stimulation and lupus-like disease development in the imiquimod (IMQ)-induced lupus model is unclear, other than the mechanism involving pDC.

Monocytes are versatile cells recruited from the circulation to the sites of inflammation, which then differentiate into dendritic cells (DC) or macrophages (MF) (12). Thus, their differentiation and role in the pathogenesis of lupus have gained increased attention (13). Conventionally, monocytes are classified into two types: “classical” Ly6C^hi^ cells in mice and CD16^-^ cells in humans, and “non-classical” Ly6C^lo^ cells in mice and CD16^+^ cells in humans (14). Classical and non-classical monocytes have a lineage relationship in which classical monocytes continuously give rise to non-classical monocytes although phenotypically different intermediate monocytes were recently reported (15, 16). Classical monocytes are involved in acute inflammatory responses. They are recruited to sites of inflammation where they extravasate and give rise to monocyte-derived DCs and MF. In contrast, non-classical monocytes patrol the endothelial surface and coordinate its repair by recruiting neutrophils as required (12). In terms of TLR7 in monocyte-related cells and their relation to SLE, monocyte-derived DCs, especially CD14^+^ cells, and their stimulation *via* TLR7 were reported to be involved in the induction of follicular helper T cells (Tfh), which are important for the differentiation of autoantibody-producing B cells (11). However, CD16^+^ monocytes accumulated in the glomerulus of kidneys in lupus nephritis patients (17) and the stimulation of TLR7 in non-classical monocytes led to inflammation that was related to the pathogenesis of lupus nephritis (18).

In this report, we investigated the involvement of monocytes and monocyte-derived cells in the development of a lupus-like disease in the IMQ-induced lupus model. By analyzing cells in the peripheral blood, lymph nodes, and kidneys at the same timepoints, we showed that numbers of Ly6C^hi^ and Ly6C^lo^ cells increased differently in terms of the developmental stage and organ location. We found that classical monocytes, non-classical monocytes, and monocyte-derived cells were involved differently in the development of SLE.

## Materials and Methods

### Mice and the Induction of Lupus-Like Disease

Female C57BL/6 mice were purchased from CLEA Japan (Tokyo, Japan), NZB mice and NZW mice were purchased from Japan SLC (Hamamatsu, Japan), and female F1 offspring were used as NZB/NZW mice. B6-EGFP mice were originally obtained from Jackson Laboratory (Bar Harbor, ME, USA) and they were bred and maintained in the animal facility at the Juntendo University School of Medicine. Mice were housed under specific pathogen-free conditions. Mice were sacrificed at 12 to 15 weeks of age unless otherwise described. All animal experiments were performed in accordance with the guidelines of laboratory animal experimentation at Juntendo University School of Medicine.

To induce lupus-like disease, mice were treated topically with 5% IMQ cream (Mochida Pharmaceutical, Tokyo, Japan). IMQ cream was applied to the ear skin but if the amount of one dose was greater than 20 mg, it was applied to the ear skin and forelegs. The dosage and duration were dependent on the experiments. Details of the treatments are described in the figure legends.

Proteinuria was analyzed using a DCA Microalbumin/Creatinine Urine Test (Siemens, Erlangen, Germany) according to the manufacturer’s instructions.

### Immunofluorescent Staining

For fluorescent staining, mouse lymph nodes and kidneys were removed after transcardial perfusion with PBS and then frozen with liquid nitrogen after being embedded in OCT compound (Sakura Finetek Japan, Tokyo, Japan). Four-micrometer cryostat sections were fixed in acetone and stained with anti-mouse IgG FITC (Southern Biotech, Birmingham, USA), anti-mouse complement C3 (MP Biomedicals, Santa Ana, CA, USA), rat anti-CD11b (Tonbo Biosciences, San Diego, CA, USA), rabbit-anti-CD3 (Abcam, Cambridge, UK) or biotin-conjugated rat anti-CD45R/B220 (BioLegend, San Diego, CA, USA). For multiple staining, primary antibodies were followed by AF 488-conjugated anti-rat IgG (Jackson ImmunoResearch, West Grove, PA, USA), rhodamine (TRITC)-conjugated donkey anti-rabbit IgG (Jackson ImmunoResearch) and Cy5-streptavidin (Thermo Fisher Scientific, Waltham, MA, USA). Staining of sections was visualized with a fluorescence microscope (BZ-X700; Keyence, Osaka, Japan).

### Immune Cell Isolation

Mice were deeply anesthetized and lymph nodes, spleens, and kidneys were removed after transcardial perfusion with PBS. Blood was drawn from the right ventricle with or without transcardial perfusion depending on the experiment. For the enzymatical digestion of lymph nodes and kidneys, collagenase (Sigma-Aldrich, St. Louis, MO, USA), and DNase (Roche, Basel, Switzerland) dissolved in RPMI 1640 medium (Thermo Fisher Scientific) supplemented with 10% fetal bovine serum, 2 mM L-glutamine, 50 U/mL penicillin, and 50 µg/mL streptomycin (all from Thermo Fisher Scientific) were used. Kidney tissues were dissociated with GentleMacs Dissociator (Miltenyi Biotec, Bergisch Gladbach, Germany), whereas lymph nodes and spleens were ground through a cell strainer. To isolate mononuclear cells, dissociated kidney tissue was suspended in 33% Percoll (GE Healthcare, Chicago, IL, USA) in PBS and overlaid on a 60% Percoll layer. After centrifugation, cells in the intermediate layer were collected and washed. For the isolation of mononuclear cells from the blood, collected blood was overlaid on a 60% Percoll layer and mononuclear cells were isolated using the same method as for kidney cells. For the isolation of splenocytes, spleens were ground through a cell strainer and erythrocytes were removed by ACK lysis. These cells were then used for flow cytometry analysis or cell sorting.

### Flow Cytometry

For flow cytometry analysis, isolated cells were pre-incubated for 10 minutes with purified anti-mouse CD16/32 (BioLegend) to block the Fc-mediated non-specific binding of antibodies. Then, cells were stained with the following antibodies: anti-CD64-PE/Dazzle 594, anti-CD115-PE/Dazzle 594, anti-CD3-PerCP/Cy5.5, anti-NK1.1-PerCP/Cy5.5, anti-Ly6G-PerCP/Cy5.5, anti-CD24-PE/Cy7, anti-CX3CR1-APC, anti-CCR5-APC, anti-Ly6C-APC/Cy7, anti-CD45-BV421, anti-I-A/I-E-BV605 (all from BioLegend), anti-CD19-PerCP/Cy5.5, anti-CD11b-AF700 (BD Biosciences, Franklin Lakes, NJ, USA), anti-CCR2-APC (R&D Systems, Minneapolis, MN, USA), and anti-F4/80-PE (Thermo Fisher Scientific) for 20 minutes on ice. After surface staining, dead cells were stained with Zombie aqua™ Fixable Viability Kit (BioLegend). For the intracellular staining of TLR7, cells were fixed and permeabilized with the BD Cytofix/Cytoperm Fixation/Permeabilization Solution Kit (BD Biosciences) according to the manufacturer’s instructions. Then, cells were stained with anti-TLR7-PE (BD Biosciences). Data were acquired on a FACS LSR Fortessa (BD Biosciences) and the percentage of each cell population and mean fluorescence intensity were analyzed using FlowJo software (TreeStar Inc, Ashland, OR, USA).

### Cell Sorting

CD11b positive cells were enriched using CD11b microbeads (Miltenyi Biotec) after the isolation of single cells from the blood, spleens, and kidneys of mice as described above. Cells were stained with the fluorochrome-conjugated antibody described above and sorted using a FACSAria Fusion cell sorter (BD Biosciences). Sorted cells were used for RNA-seq, quantitative real-time polymerase chain reaction (qRT-PCR), or cell transfer.

### RNA-Seq Analysis

Total RNA was isolated from sorted cells using an RNeasy Micro Kit (Qiagen, Hilden, Germany). RNA-seq libraries were generated with the Ovation SoLo RNA-Seq System, Mouse kit (NuGEN, Redwood City, CA, USA) using 5 ng of total RNA. The cDNA libraries were sequenced by 50-base single-read sequencing on an Illumina HiSeq 2500 sequencer (Illumina, San Diego, CA, USA). The sequencing run and base call analysis were performed according to the HiSeq 2500 System Guide with TruSeq SBS kit v3-HS. After sequencing, raw sequence data were generated with processing by CASAVA-1.8.4 version RTA 1.17.20.0. Reads were mapped to the mm10 genome with tophat2. Normalized FPKM values and differential gene expression analyses were generated with Cuffdiff and CummeRbund. Q-values (Benjamini-Hochberg correction) lower than 0.05 were considered significant. Gene ontology enrichment analysis was performed using metascape (http://metascape.org). Heatmaps were generated using Heatmapper (http://www.heatmapper.ca/).

### Stimulation of Monocytes and RNA Preparation for qRT-PCR

Sorted monocytes from the peripheral blood of NZB/NZW mice were placed in a round bottom 96-well plate at 3×10^4^ cells per well in RPMI medium supplemented with fetal bovine serum, L-glutamine, penicillin, and streptomycin as described above. Then, cells were stimulated with IMQ (R837) (5 µg/ml; *In vivo*Gen, San Diego, CA, USA) or 2′3′-cGAMP (25 µg/ml; *In vivo*Gen) for 2 h at 37°C in a 5% CO_2_ incubator. After incubation, RNA was obtained using an RNeasy Micro Kit.

### qRT-PCR Analysis

cDNA was prepared from total RNA by reverse transcription with ReverTra Ace qPCR RT Master Mix (Toyobo, Osaka, Japan). Fast SYBR Green Master Mix (Thermo Fisher Scientific) was used for the amplification of *Itga9, Itgad, Mertk, Vcam1, Cxcl13, Ccl5, Il6*, and *Il10* according to the manufacturer’s instructions. Results were normalized to *Gapdh*. The following primers were used: *Itga9* forward: 5′-TGTTTTGGCCTGTGCCCATC-3′; *Itta9* reverse: 5′-GGGAATCAGCACCTTGCCTT-3′; *Itgad* forward: 5′-TCCAGAAAGTGGTAGACAGCAA-3′; *Ittad* reverse: 5′-GAGTGTTGTAGTGGCAACCTG-3′; *Mertk* forward: 5′-AGCTGGCATTTCATGGTGGA-3′; *Mertk* reverse: 5′-CTGCACACTGGCTATGCTGA-3′; *Vcam1* forward: 5′-AGAACTACAAGTCTACATCTCTCCC-3′; *Vcam1* reverse: 5′-GTCACAGCACCACCCTCTT-3′; *Cxcl13* forward: 5′-CCACCTCCAGGCAGAATGAG-3′; *Cxcl13* reverse: 5′-TGGGCTTCCAGAATACCGTG-3′; *Ccl5* forward: 5′-CAGTCGTGTTTGTCACTCGAA-3′; *Ccl5* reverse: 5′-AGAGCAAGCAATGACAGGGA-3′; *Il6* forward: 5′-CACTTCACAAGTCGGAGGCT-3′; *Il6* reverse: 5′-CTGCAAGTGCATCATCGTTGT-3′; *Il10* forward: 5′-CTTTAAGGGTTACTTGGGTTGCC-3′; *Il10* reverse: 5′-TTCTGGGCCATGCTTCTCTG-3′; *Gapdh* forward: 5′-GCAAGGACACTGAGCAAGAGA-3′; and *Gapdh* reverse: 5′-AGGCCCCTCCTGTTATTATG-3′. TaqMan™ Gene Expression Assay was used for *Ifna4* (Mm00833969_s1), *Ifna5* (Mm00833976_s1), and *Ifnb1* (Mm00439546_s1). Results were normalized to *Gapdh* (Mm99999915_g1). THUNDERBIRD^®^ Probe qPCR Mix (Toyobo) was used for amplification according to the manufacturer’s instructions. All qRT-PCR were performed with a 7500 Fast Real-Time PCR System (Thermo Fisher Scientific). Fold-changes in gene expression were calculated using the 2^−ΔΔCt^ method.

### Monocyte Transfer

Single cells were obtained from the peripheral blood and spleens of IMQ induced or control NZB/NZW mice. Cell sorting was conducted as described above and monocytes were isolated. Monocytes were labeled with CellTrace™ Cell Proliferation Kits (Thermo Fisher Scientific) according to the manufacturer’s instructions. Cells were suspended in PBS and 3–5 ×10^5^ cells were transferred into recipient mice through the tail vein.

### Bone Marrow Transplantation

Using an MBR-1505R2 system (Hitachi Power Solutions, Hitachi, Japan), EGFP^-/-^ mice were irradiated twice with 600 rad 4 h apart. Before irradiation, mice kidneys were protected by covering the abdominal part with a self-made protector consisting of a 0.6 mm thick lead band. This was used to prevent tissue injury and keep resident cells in the tissues (19). A day after irradiation, 1×10^6^ total bone marrow cells harvested from the femurs of EGFP^+/-^ mice were suspended in PBS and transferred into irradiated recipient mice through the tail vein. Three weeks after BM reconstruction, chimerism was confirmed by the FACS analysis of peripheral blood.

### Statistical Analysis

Statistical analyses (except for RNA-seq) were performed using Prism software (Graphpad, La Jolla, CA, USA). Significance was determined by Student’s *t*-test or one-way ANOVA followed by the post-tests described in the figure legends.

## Results

### Different Spatial and Temporal Increase of Monocyte-Like Cells Induced by IMQ Is Accompanied by the Development of Nephritis

**Figure 1:**
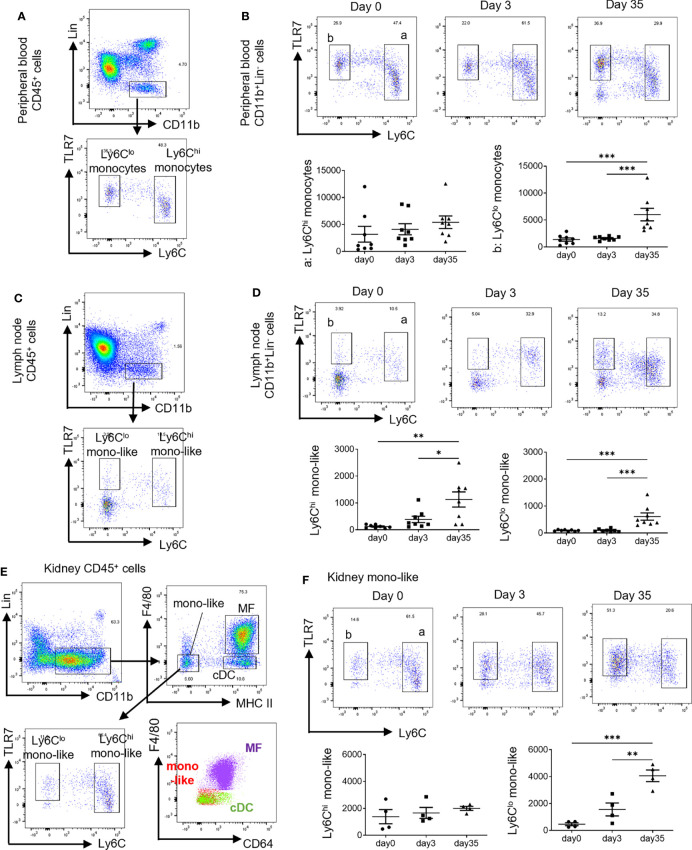
Different spatial and temporal increases in monocyte-like cells by treatment with IMQ. In the short-term analysis, C57BL/6 mice were treated with 40 mg of IMQ on days 0 and 2, then sacrificed on day 3. In the long-term analysis, mice were treated with 40 mg of IMQ three times a week for 35 days. **(A)** Monocytes were identified as CD11b^+^Lin^-^ cells in the peripheral blood. Ly6C^hi^ monocytes and Ly6C^lo^ monocytes are shown relative to the expression of TLR7. **(B)** The change of peripheral Ly6C^hi^ and Ly6C^lo^ monocytes at 3 and 35 days after the application of IMQ. **(C)** Among CD11b^+^Lin^-^ cells in the lymph nodes, Ly6C^hi^ monocyte-like cells and Ly6C^lo^ monocyte-like cells were identified based on the expressions of Ly6C and TLR7. **(D)** The change of cervical lymph node monocyte-like cells 3 and 35 days after the application of IMQ. **(E)** Kidney monocyte-like cells were identified as CD11b^+^Lin^-^F4/80^-^MHCII^lo^ cells. **(F)** Changes in kidney monocyte-like cells at 3 and 35 days after the application of IMQ. **(B, D, F)** show the combined data of two experiments. Numbers in the graph represent the number of cells per 100,000 CD45^+^ cells. Symbols represent individuals and horizontal lines indicate the mean and SEM. *P < 0.05, **P < 0.01, and ***P < 0.001 by one way ANOVA and *post hoc* Tukey’s multiple comparison test. IMQ, imiquimod; Lin, lineage marker (CD3, CD19, Ly6G, NK1.1); mono-like, monocyte-like cells; MF, macrophages; cDC, conventional dendritic cells.

Monocytes can be identified as CD11b^+^CD115^+^CX3CR1^+^cells and roughly classified into two types: Ly6C^hi^ and ly6C^lo^ monocytes (12, 15). In the steady state, peripheral blood CD11b^+^ cells among lineage marker (Lin: CD3, CD19, Ly6G, NK1.1) negative cells were mostly composed of monocytes that expressed CD115 and CX3CR1 (**Supplementary Figures 1A, B**). Considering the importance of TLR7 signals in monocytes and monocyte-related cells in the pathogenesis of SLE (11, 18), we focused on the expression of TLR7 in relation to monocytes. Ly6C^hi^ monocytes expressed TLR7 at a low to intermediate level and Ly6C^lo^ monocytes expressed intermediate levels of TLR7 (**Figure 1A**). There was no significant change in the number of peripheral blood monocytes in mice treated with short-term (3 days) topical IMQ. In contrast, long-term (35 days) IMQ treatment increased the number of peripheral blood Ly6C^lo^ monocytes (**Figure 1B**). At the same time, we investigated the cervical lymph nodes, the draining lymph nodes of IMQ-treated ears. In the steady state, most CD11b^+^Lin^-^ cells in the lymph nodes were Ly6C^lo^TLR7^-^ (**Figure 1C** and **Supplementary Figure 1C**), whereas cells with expressions of Ly6C and TLR7 similar to that of peripheral blood monocytes (shown as Ly6C^hi^ mono-like and Ly6C^lo^ mono-like) were scarce. Ly6C^hi^ monocyte-like cell numbers tended to increase with short-term IMQ treatment and were significantly increased with long-term IMQ treatment (**Figure 1D**). Ly6C^lo^ monocyte-like cell numbers also increased with the long-term treatment.

For the kidneys, we had to exclude MF from CD11b^+^Lin^-^ cells before the analysis of monocytes because there were high numbers of TLR7-expressing MF in the FACS gate. In the steady state, CD11b^+^Lin^-^ cells in kidneys were roughly classified into three subsets by the expression of F4/80 and MHC class II (**Figure 1E** and **Supplementary Figure 1D**). In the kidneys, MHC II^hi^ cells were divided into F4/80^+^CD64^+^ MF and F4/80^-^CD64^-^ DC (20). MHC II^lo^ cells were considered to include monocyte-derived cells (21). Because F4/80 is a marker of MF, MHC II^lo^ F4/80^-^ cells are considered monocyte-like cells that comprise Ly6C^hi^ and Ly6C^lo^ cells based on the expressions of Ly6C and TLR7, which were observed in blood monocytes (**Figure 1E**). Ly6C^hi^ monocyte-like cell numbers did not increase with short-term nor long-term treatment, although long-term treatment increased Ly6C^lo^ monocyte-like cell numbers (**Figure 1F**).

**Figure 2:**
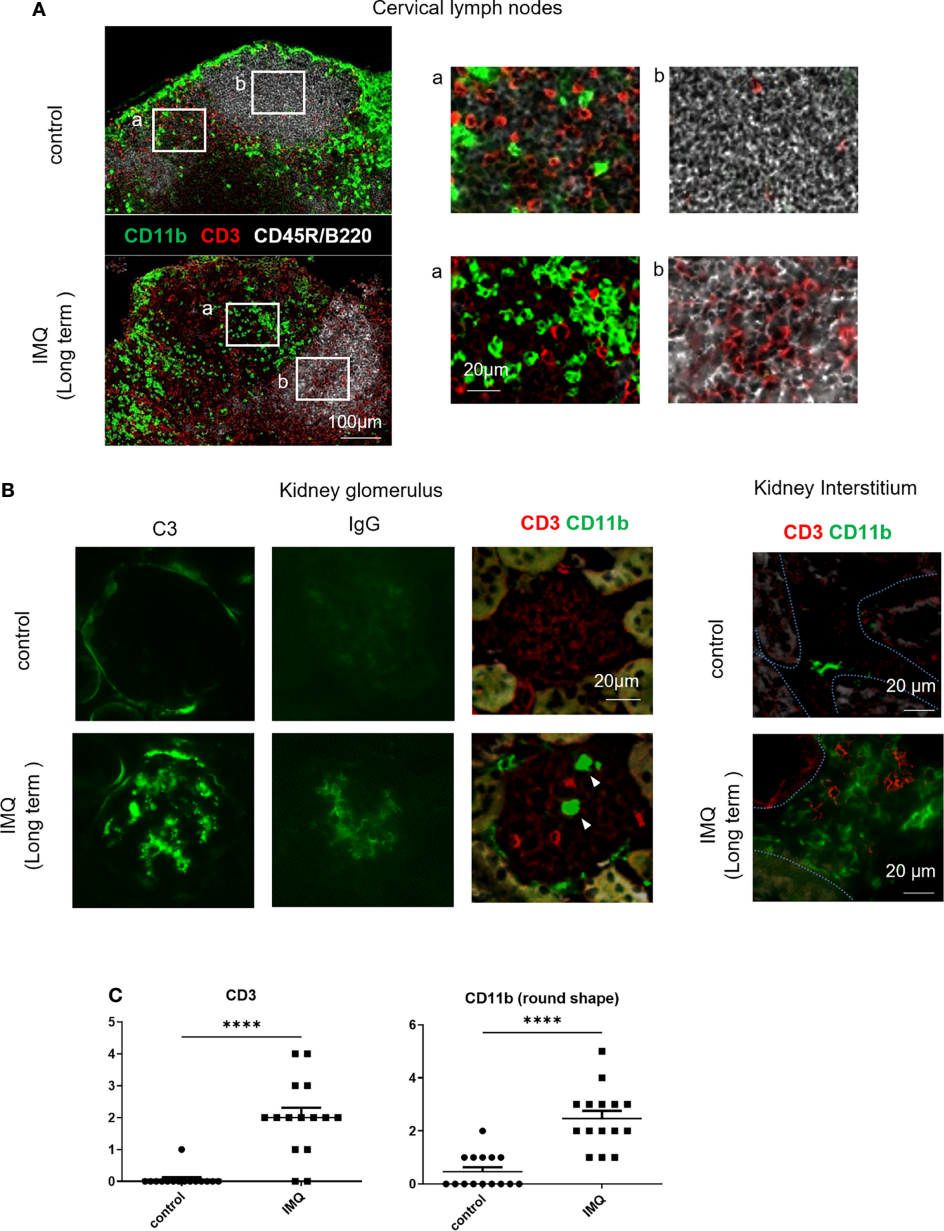
Immunofluorescent staining of cervical lymph nodes and kidneys of IMQ-induced lupus mice. Mice were treated with 40 mg of IMQ three times a week for 5 weeks. Frozen sections were stained with the indicated antibodies. **(A)** Immunofluorescent staining of cervical lymph nodes from control and IMQ-induced lupus mice. **(B)** Immunofluorescent staining of kidneys from control and IMQ-induced lupus mice. Arrowheads indicate round cells in the glomeruli. Dashed lines in the right panel outline the tubular epithelium. **(C)** CD3^+^ and CD11b^+^ round cells in five representative glomeruli were counted twice from three mice. Cell number per glomerulus is shown. **(A, B)** are representative images of two experiments (n=3). ****P < 0.0001 by Student’s *t*-test. T cell zones (a) and follicles (b) were magnified.

These observed changes of monocytes and monocyte-like cells by long-term treatment were accompanied by the development of proteinuria, which indicates the development of nephritis (**Supplementary Figure 2A**). Immunofluorescent staining of cervical lymph nodes revealed monocyte-like round shaped CD11b^+^ cells mainly in the T cell zones and high numbers of CD3^+^ cells in the follicles of long-term treated mice indicating germinal center formation (**Figure 2A**). Deposition of immune-complex in the glomerulus shown by the positive staining for complement C3 and IgG indicated a lupus-like pathogenesis in the nephritis (**Figure 2B**). Increased CD11b^+^ and CD3^+^ cell numbers were observed in the glomerulus and interstitium (**Figures 2B, C**). Increased CD11b^+^ cells can be explained by the increase of Ly6C^lo^ monocytes (round cells) and the upregulated expression of CD11b in MF in the interstitium. The CD11b expression of MF was higher in IMQ-induced lupus mice than in controls (**Supplementary Figure 2B**).

### Monocyte-Like Cells and MF in the Kidneys Are Replaced by Peripheral Blood Cells After the Long-Term Application of IMQ

**Figure 3:**
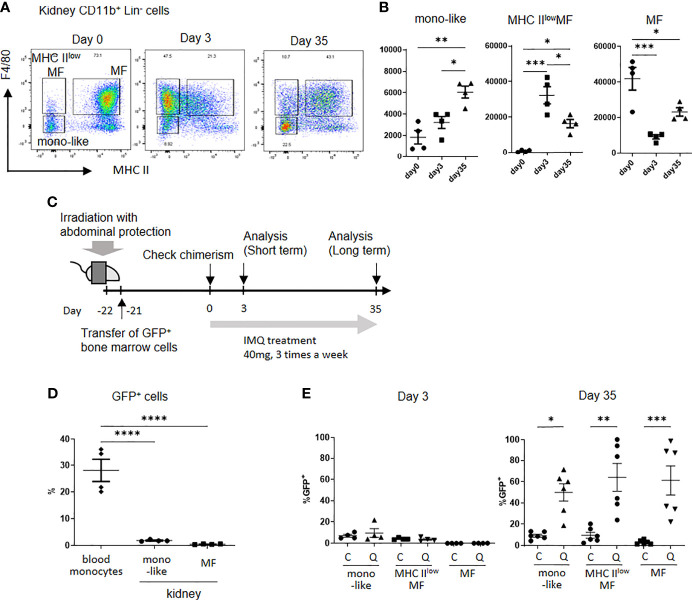
Kidney monocyte-like cells and MF are replaced by peripheral blood cells after the long-term application of IMQ. **(A)** Representative plot of kidney CD11b^+^Lin^-^ cells from control (left), short-term IMQ treatment (middle), and long-term IMQ treatment (right) mice. In the short-term analysis, mice were treated with 40 mg of IMQ on days 0 and 2 then sacrificed on day 3. In the long-term analysis, mice were treated with 40 mg of IMQ three times a week for 35 days. **(B)** The change in kidney monocyte-like, MHC II^low^MF (MHC II^lo^F4/80^+^), and MF (MHC II^hi^F4/80^+^) cells at 3 and 35 days after the application of IMQ. **(C)** Kidney-protected GFP^-^ mice were irradiated and GFP^+^ cells were transferred. Three weeks after transplantation, treatment with topical IMQ was started. **(D)** Percentages of GFP positive cells 21 days after bone marrow transplantation. Partial bone marrow chimera and protection of kidney resident cells were confirmed. **(E)** Percentages of relative GFP^+^ cells in control **(C)** and IMQ-treated mice (Q) at days 3 and day 35 after treatment. The percentages were adjusted according to the blood GFP^+^ cell ratio. **(B, D, E)** show the combined data of two experiments. Symbols represent individuals and horizontal lines indicate the mean and SEM. In **(B)**, the number in the graph represents the number of cells per 100,000 CD45^+^ cells. *P < 0.05, **P < 0.01, ***P < 0.001, and ****P < 0.0001 by one way ANOVA and *post hoc* Tukey’s multiple comparison test. mono-like: monocyte-like cells, MHC II^low^MF: MHC II^lo^ macrophage-like cells, MF: macrophages.

Analysis of monocyte-like cells and MF in kidneys under the treatment of IMQ, showed short-term IMQ treatment decreased MF numbers. In contrast, F4/80^+^ cells expressing a low level of MHC II (MHC II^low^MF) were increased at the same time. The long-term application of IMQ decreased MHC II^low^MF and increased MF. The number of monocyte-like cells, especially Ly6C^lo^ mono-like cells, was increased in the long-term treated mice (**Figures 1F**, **3A, B**). Although MF in the kidneys are usually not replenished by circulating monocytes under steady state conditions, they are replaced by monocyte-derived cells if there is inflammation in the kidneys (21). To investigate whether CD11b^+^Lin^-^ cells in the kidneys of IMQ treated mice were replaced by circulating monocytes, we analyzed the dynamics of CD11b^+^ cells in the kidneys using a bone marrow chimera generated by the transplantation of B6-EGFP^+/-^ bone marrow cells into B6-EGFP^-/-^ mice (**Figure 3C**). Mice were irradiated with a lead band that covered the abdomen to protect kidney resident cells. Approximately 30% of circulating monocytes became GFP positive, whereas 1%–2% of monocyte-like cells and 0.5% of MF in the kidneys were GFP positive 3 weeks after bone marrow transplantation (**Figure 3D**). When IMQ was applied over the short-term, there was no increase in GFP positive cells in kidney monocyte-like cells (control 7% vs IMQ 9%), MHC II^low^MF (control 2% vs IMQ 4%), or MF (control 0% vs IMQ 0%) (**Figure 3E**). However, with the long-term application, approximately 50%–60% of monocyte-like cells, MHC II^low^MF, and MF were replaced by circulating monocytes when normalized by the percentages of GFP positive cells in the circulating monocytes (monocyte-like cells: control 9% vs IMQ 50%; MHC II^low^MF: control 10% vs IMQ 66%; MF: control 3% vs IMQ 61%) (**Figure 3E**). These results indicate the short-term increase of MHC II^low^MF did not result from replenishment by circulating monocytes but probably as a result of a phenotypical change of MF in the kidneys. Monocyte-like cells, MHC II^low^MF, and MF observed in long-term treated mice originated, at least in part, from infiltrated circulating hematopoietic cells.

### RNA-Seq Analysis Reveals the Upregulation of Adhesion-Related Genes in Ly6C^lo^ Monocytes and Inflammatory Features of Kidney Monocyte-Like Cells

These findings indicated that long-term IMQ treatment increased Ly6C^lo^ monocytes in the circulation and Ly6C^lo^ monocyte-like cells in affected organs such as kidneys. The monocyte-like cells in the kidneys partially originated from the infiltration of circulating hematopoietic cells. To characterize these monocyte-like cells, we conducted RNA-seq analysis of peripheral blood monocytes and monocyte-like cells in the kidneys. Monocytes and monocyte-like cells were identified as CD11b^+^Lin^-^MHC II^lo^CD64^-^F4/80^-^CX3CR1^+^ cells (**Supplementary Figure 3**). CD115 was not a suitable marker because of its internalization during the cell isolation process (22). Thus, we used CX3CR1 as a substitute marker for monocytes and monocyte-like cells after the exclusion of MF and DC by excluding CD64^+^, F4/80^+^, and MHC II^hi^ cells. Isolated cells were indicated using the following abbreviations: B-Ly6C^hi^: blood Ly6C^hi^ monocytes; B-Ly6C^lo^: blood Ly6C^lo^ monocytes; K-Ly6C^lo^: kidney Ly6C^lo^ monocyte-like cells. RNA was obtained from these cells and analyzed. The number of reads per sample ranged from 16 to 22 million, and 96%–97% were successfully mapped onto the mm10 genome. Among them, we found 8542 differentially expressed genes (DEG).

**Figure 4:**
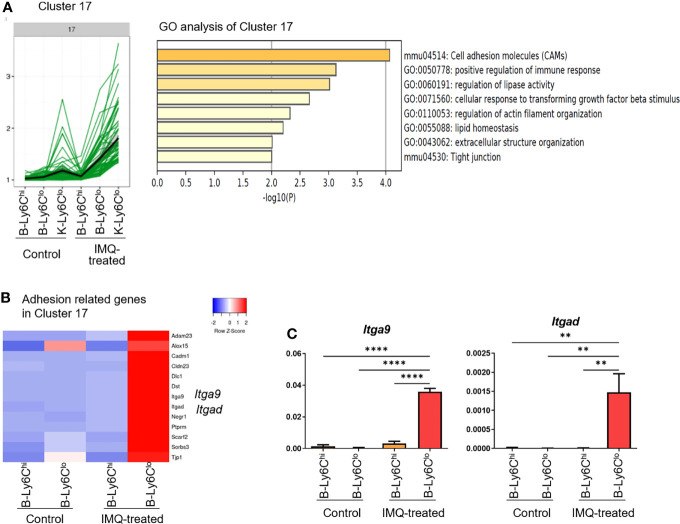
RNA-seq analysis reveals the upregulation of adhesion-related genes in peripheral Ly6C^lo^ monocytes of IMQ-induced lupus mice. **(A)** In the RNA-seq analysis, cluster 17 among 20 clusters was characterized by genes highly expressed in Ly6C^lo^ monocyte-like cells in the kidneys and Ly6C^lo^ blood monocytes in IMQ-treated mice. Gene ontology analysis revealed adhesion-related genes were enriched in this cluster. **(B)** Thirteen genes with the gene ontology term “cell adhesion” in cluster 17. These genes were upregulated in blood Ly6C^lo^ monocytes from IMQ-treated mice. **(C)** qRT-PCR analysis of *Itga9* and *Itgad* in blood monocytes. RNA-seq and q-RT PCR results are of pooled samples from four groups of mice each analyzed individually (16 control or 5 IMQ-treated mice were used for each pooled sample in the RNA-seq analysis. Ten control or 3 IMQ-treated mice were used for each pooled sample in the q-RT PCR analysis). In the qRT-PCR analysis, the y-axis shows the fold change expression in comparison with the expression of *Gapdh*. Bar and horizontal lines indicate the mean and SEM. **P < 0.01 and ****P < 0.0001 by one way ANOVA and *post hoc* Dunnett’s multiple comparison test. B-Ly6C^hi^; blood Ly6C^hi^ monocytes, B-Ly6C^lo^; blood Ly6C^lo^ monocytes, K-Ly6C^lo^; kidney Ly6C^lo^ monocyte-like cells.

**Figure 5:**
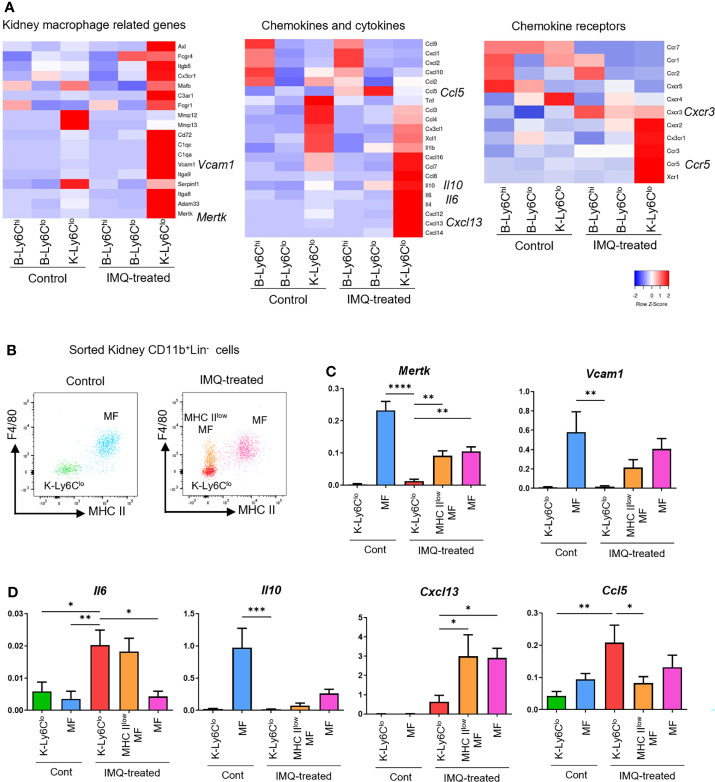
Gene expression features of monocytes, monocyte-like cells, and MF in IMQ-induced lupus mice. **(A)** RNA-seq analysis of blood monocytes and kidney monocyte-like cells in the controls and IMQ-treated mice. **(B)** Plot of sorted monocyte-like cells and MF from control and IMQ-treated mice. Each cell type was sorted from kidney CD11b^+^Lin^-^ cells as follows. K-Ly6C^lo^ = F4/80^-^MHCII^lo^Ly6C^lo^CX3CR1^+^ cells; MHC II^low^MF = MHC II^lo^F4/80^+^cells; MF = MHC II^hi^F4/80^+^cells. **(C, D)** qRT-PCR analysis of sorted kidney cells from control and IMQ-treated mice. RNA-seq and qRT-PCR results are of pooled samples from four groups of mice each analyzed individually (16 control or 5 IMQ-treated mice were used for each pooled sample in the RNA-seq analysis. Ten control or 3 IMQ-treated mice were used for each pooled sample in the qRT-PCR analysis). In the qRT-PCR analysis, the y-axis shows the fold change expression in comparison with the expression of *Gapdh*. Bar and horizontal lines indicate the mean and SEM. *P < 0.05, **P < 0.01, ***P < 0.001, and ****P < 0.0001 by one way ANOVA and *post hoc* Dunnett’s multiple comparison test. B-Ly6C^hi^, blood Ly6C^hi^ monocytes; B-Ly6C^lo^, blood Ly6C^lo^ monocytes; K-Ly6C^lo^, kidney Ly6C^lo^ monocyte-like cells. MHC II^low^MF, MHC II^lo^ macrophage-like cells; MF, macrophages.

Using the k-means clustering method, DEG were classified into 20 clusters (**Supplementary Figure 4**). Clusters 16, 17, and 18 were characterized by the genes that were most upregulated in IMQ-induced K-Ly6C^lo^ (**Supplementary Figure 4**). Among them, cluster 17 was characterized by genes also upregulated in IMQ-induced B-Ly6C^lo^. In this cluster, an enrichment of genes related to cell adhesion was observed (**Figure 4A**). Among the 64 genes in cluster 17, 13 genes had the gene ontology name “cell adhesion” and when compared among peripheral blood monocytes, these genes were the most upregulated in IMQ-induced B-Ly6C^lo^ (**Figure 4B**). The upregulated genes included genes of integrins such as *Itga9* and *Itgad*, which were also confirmed by qRT-PCR analysis (**Figure 4C**). Among the genes in clusters 16, 17, and 18, were those characteristic of MF in the kidneys including *Adam33* (Cluster 16), *Vcam1*, *Mertk*, *C1qa*, *Itga9* (Cluster 17), *CD72*, *C1qc*, and *Itga8*, (Cluster18) (23). IMQ-induced K-Ly6C^lo^ showed the upregulation of MF-related genes compared with monocytes and monocyte-like cells (**Figure 5A**). In terms of chemokines and cytokines, the expression of *CCl5* was generally higher in IMQ-treated mice than controls and it was noteworthy that *Ccr5*, the CCL5 receptor gene, was upregulated in IMQ-induced K-Ly6C^lo^. The upregulation of several genes encoding cytokines and chemokines including *Il10*, *Il6*, and *Cxcl13* were also observed in IMQ-induced K-Ly6C^lo^. Furthermore, *Cxcr3* was upregulated in monocytes and monocyte-like cells from IMQ-treated mice by RNA-seq analysis, which was in accordance with the recently reported importance of the IP-10/CXCR3 axis in human lupus nephritis (24).

To assess whether IMQ-induced K-Ly6C^lo^ were macrophage-like and inflammatory, we analyzed kidney monocyte-related cells and assessed macrophage-related genes and characteristic cytokine and chemokine genes that were upregulated in IMQ-induced K-Ly6C^lo^ by RNA-seq analysis. MHCII^low^MF, MF, and K-Ly6C^lo^ were sorted from kidney CD11b^+^Lin^-^ cells of control and IMQ-treated mice (**Figure 5B**) and gene expressions were analyzed by qRT-PCR. *Mertk* and *Vcam1*, characteristic macrophage-specific genes were upregulated in IMQ-induced K-Ly6C^lo^ by RNA-seq analysis but at a lower level than that in MF (**Figure 5C**). Although *Il6* and *Il10* appeared upregulated in IMQ-induced K-Ly6C^lo^ by RNA-seq analysis, PCR analysis revealed that the expression of *Il10* in K-Ly6C^lo^ was minimal compared with that in control MF (**Figure 5D**). *Il6* was upregulated in IMQ-induced K-Ly6C^lo^ compared with MF. RNA-seq analysis showed the upregulation of *Cxcl13* in IMQ-induced K-Ly6C^lo^ from IMQ-treated mice and PCR analysis revealed its expression was even higher in MHCII^low^MF and MF from IMQ-treated mice. The CCL5/CCR5 axis was previously reported to be involved in the recruitment of Ly6C^lo^ monocytes to tissues (25). *Ccl5* was upregulated in IMQ-induced K-Ly6C^lo^ and lower in MF (**Figure 5D**).

These results indicated that the upregulated adhesion-related genes in IMQ-induced B-Ly6C^lo^ were likely related to the infiltration of monocytes to the kidneys. Upregulated *Ccl5* in monocytes and monocyte-like cells in IMQ-treated mice and upregulated *Ccr5* in kidney monocyte-like cells by RNA-seq data suggested the involvement of the CCL5/CCR5 axis in the infiltration of monocytes into the kidneys. Upregulated *Il6* in IMQ-induced K-Ly6C^lo^ indicated the inflammatory features of these cells. The expressions of *Il6* and *Il10* indicated that inflammatory features were decreased related to the differentiation of monocytes to MF. Although MF in IMQ-treated mice had similar gene expression patterns to the control MF, *Cxcl13* expression in MF was limited to IMQ-treated mice, suggesting its importance in the disease pathogenesis.

### IMQ-Induced Conditions Are Characterized by the Infiltration of Ly6C^lo^ Monocytes Into Tissues

The upregulated expressions of adhesion-related genes in Ly6C^lo^ monocytes of IMQ-treated mice demonstrated by RNA-seq analysis suggested they were more prone to infiltrate into tissues. To clarify the infiltrating ability of monocytes into tissues, we performed an adoptive transfer experiment using sorted monocytes from IMQ-induced lupus mice.

NZB/NZW mice are a well-known model of the spontaneous development of lupus-like disease. In this model, along with the development of disease, Ly6C^lo^ monocytes and Ly6C^lo^ monocyte-like cells were increased spontaneously in the blood and kidneys of aged mice (**Supplementary Figure 5A**). Similar to IMQ-induced lupus mice, Ly6C^hi^ monocytes tended to be increased in the lymph nodes whereas Ly6C^lo^ monocytes and monocyte-like cells were dominant in the peripheral blood and kidneys. This increase of monocytes and monocyte-like cells was promoted by the application of IMQ to the ears of NZB/NZW mice, and the early development of nephritis was indicated by the development of proteinuria (**Supplementary Figures 2A** and **5A–D**). Thus, the advantage of a shorter time course of IMQ-induced disease development in NZB/NZW mice led us to use this model to analyze the function of monocytes in further experiments.

**Figure 6:**
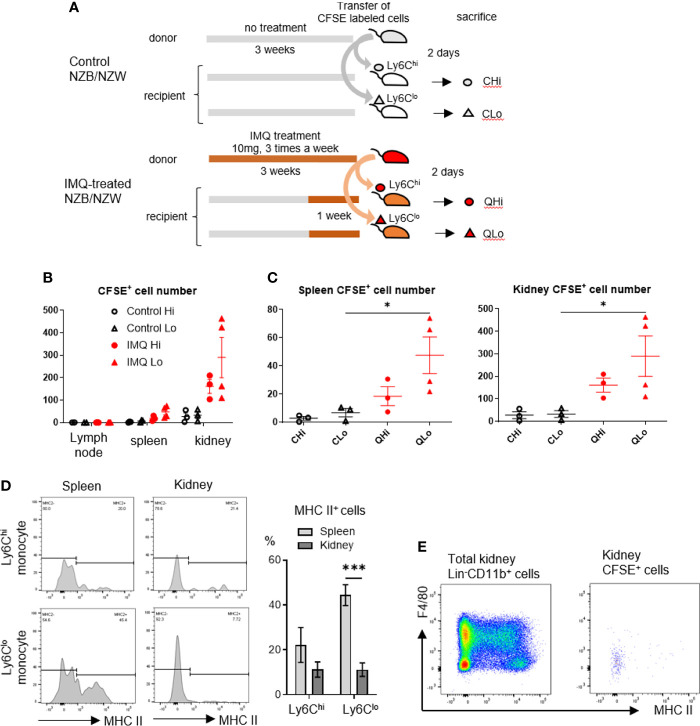
Ly6C^lo^ monocytes from IMQ-treated mice are more prone to infiltrate into tissues. **(A)** Sorted monocytes from control or IMQ-treated (10 mg, three times a week for 3 weeks) NZB/NZW mice were transferred to recipient control or IMQ-treated mice and tissues were analyzed 2 days after transfer. **(B)** Transferred CFSE labeled monocytes recovered from tissues were analyzed. **(C)** Transferred CFSE labeled monocytes recovered from spleens and kidneys were analyzed. **(D)**: CFSE^+^Ly6C^lo^ monocytes recovered from spleens showed upregulated MHC class II whereas most transfused Ly6C^lo^ monocytes in kidneys remained MHC class II negative. **(E)** Transferred Ly6C^lo^ monocytes in kidneys maintained F4/80^-^MHC II^lo^ monocyte-like surface markers. In A and B, numbers of CFSE positive cells per 1×10^5^ CD45^+^ cells are shown. Numbers were adjusted according to the transferred cell number that was normalized to 1×10^5^. **(B–D)** show the combined data of four experiments. Symbols represent individuals and horizontal lines indicate the mean and SEM. *P < 0.05 and ***P < 0.001 by one way ANOVA and *post hoc* Bonferroni’s Multiple Comparison Test.

In the adoptive transfer experiment, monocytes were isolated from the peripheral blood and spleens because these cells are identical (26). Ly6C^hi^ and Ly6C^lo^ monocytes (CD11b^+^Lin^-^F4/80^-^MHC II^lo^CD115^+^) from control NZB/NZW mice and IMQ-treated NZB/NZW mice were labeled by CFSE and transferred to recipient mice. Two days after transfer, the cervical lymph nodes, spleens, and kidneys of recipient mice were analyzed (**Figure 6A**). We recovered higher numbers of CFSE^+^ transferred Ly6C^lo^ monocytes from the spleens and kidneys of IMQ-treated mice compared with control mice although there was a negligible infiltration of these cells to the lymph nodes (**Figures 6B, C**). Transferred Ly6C^lo^ monocytes in spleens upregulated MHC class II although monocytes recovered in the kidneys maintained their low expression of MHC II. Ly6C^hi^ monocytes in spleens showed a similar tendency for the high expression of MHC class II compared with those in the kidneys, although the difference was not statistically significant (**Figure 6D**). Ly6C^lo^ monocytes recovered in the kidneys maintained their low expression of F4/80. Thus, they maintained F4/80^-^MHC II^lo^ monocyte-like surface markers in the short-term after infiltrating into the kidneys (**Figure 6E**). These findings suggest that Ly6C^lo^ monocytes in IMQ-treated mice were more prone to infiltrate into tissues and that they underwent different phenotypical changes dependent on the tissues they infiltrated.

### Ly6C^hi^ Monocytes and Ly6C^lo^ Monocytes Have Different Inflammatory Features Upon Stimulation

**Figure 7:**
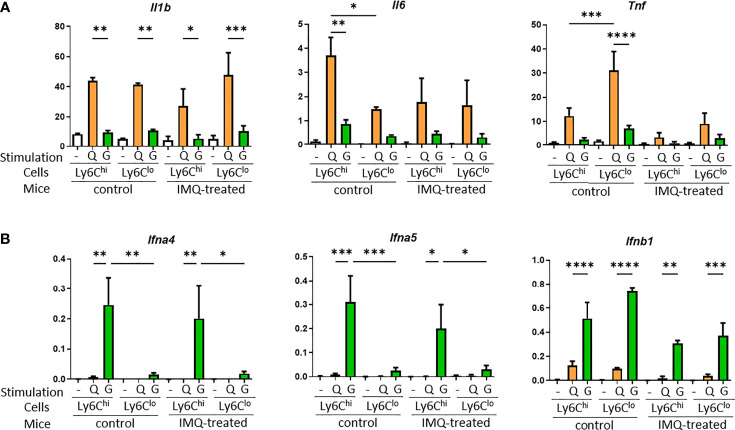
Proinflammatory cytokine and type I interferon expression in monocytes upon stimulation with TLR7 or STING. Sorted monocytes from control or IMQ-treated NZB/NZW mice were stimulated by IMQ or the STING agonist, 2′3′-cGAMP. **(A)** Proinflammatory cytokines were upregulated by IMQ stimulation compared with 2′3′-cGAMP. **(B)** Type I interferon genes were upregulated by stimulation with 2′3′-cGAMP. Expression of IFN–α genes were mostly limited to Ly6C^hi^ monocytes. -, no stimulation; Q, IMQ stimulation; G, 2′3′-cGAMP stimulation. Bar and horizontal lines indicate the mean and SEM. *P < 0.05, **P < 0.01, ***P < 0.001, and ****P < 0.0001 by one way ANOVA and *post hoc* Bonferroni’s Multiple Comparison Test.

To further analyze the different features of Ly6C^hi^ and Ly6C^lo^ monocytes in the lupus-like inflammatory environment, peripheral blood monocytes from control and IMQ-treated NZB/NZW mice were sorted and stimulated *in vitro*. Type I interferons are important for the pathogenesis of SLE and serum from SLE patients induced type I interferon-stimulated genes dependent on the agonist of stimulator of interferon genes (STING) activity (27). A study of human SLE reported IFN-α production by monocytes stimulated by 2′3′-cyclic-GMP-AMP (cGAMP), an agonist of STING, positively correlated with SLE disease activity (28). Therefore, we used IMQ and 2′3′-cGAMP to stimulate monocytes and analyzed the gene expressions of proinflammatory cytokines and type I interferons. Upon IMQ stimulation, Ly6C^hi^ and Ly6C^lo^ monocytes tended to upregulate *Il1b*, *Il6*, and *Tnf* compared with 2′3′-cGAMP. Ly6C^hi^ monocytes showed a higher upregulation of *Il6* than Ly6C^lo^ monocytes and Ly6C^lo^ monocytes showed a higher upregulation of *Tnf* in accordance with a previous report of human classical CD14^+^ and non-classical CD14^lo^ non-classical monocytes (10). These features were lost and responses tended to be reduced in monocytes from IMQ-treated mice (**Figure 7A**). However, type 1 IFN genes were upregulated in monocytes when stimulated with 2′3′-cGAMP but not IMQ. Although *Ifnb1* was upregulated similarly in Ly6C^hi^ and Ly6C^lo^ monocytes, IFN-α genes (*Ifna4* and *Ifna5*) were upregulated only in Ly6C^hi^ monocytes when stimulated with 2′3′-cGAMP (**Figure 7B**). This higher response of IFN-α genes in Ly6C^hi^ monocytes to 2′3′-cGAMP correlated with the higher expression of *Tmem173*, which encodes STING protein (mean FPKM of *Tmem173* was 59.78 in Ly6C^hi^ monocytes and 22.62 in Ly6C^lo^ monocytes by RNA-seq analysis).

Taken together, monocytes and monocyte-related cells are likely to be important for the development of lupus pathogenesis. They possess different spatial and temporal roles. The application of IMQ indicated Ly6C^hi^ monocytes responded in the early phase and highly expressed IFN-α genes after the stimulation of STING, a DNA sensor. In contrast, Ly6C^lo^ cells were markedly increased in the late phase. They showed a greater infiltration into tissues, which might be related to the upregulation of adhesion-related molecules when they are in circulation. Furthermore, the gene expressions of proinflammatory cytokines such as *Il6* and chemokines such as *Cxcl13* and *Ccl5* in Ly6C^lo^ monocyte-like cells in the kidneys indicate they are likely to be involved in the inflammatory response.

## Discussion

In the present study, we investigated the dynamic change of immune cells in the development of lupus-like disease in an IMQ-induced lupus model focusing on monocytes and monocyte-derived cells.

Ly6C^hi^ monocyte-like cell numbers were increased in the cervical draining lymph nodes starting from the initial phase, which was in accordance with an acute inflammatory response. In the late phase, Ly6C^lo^ monocytes and monocyte-like cell numbers were increased throughout the body. Increased atypical monocytes were previously reported in aged NZB/NZW mice and other lupus models (29, 30). Thus, an increase in Ly6C^lo^ monocytes is a common feature shared among lupus models. Interestingly, Ly6C^hi^ cells continued to increase in the draining lymph nodes whereas an increase in Ly6C^hi^ cells was not observed in the affected kidneys throughout the entire disease course. From these results, we hypothesized that Ly6C^hi^ and Ly6C^lo^ cells have different temporal and spatial roles in the pathogenesis of SLE. Increased numbers of Ly6C^hi^ cells in lymphoid organs are likely to be related to the induction of autoimmunity as previously reported for monocyte-derived DC (moDC) during Tfh induction, a mechanism mediated by the stimulation of TLR7 expressed by moDC (11).

Another indication of the role of Ly6C^hi^ cells in the development of autoimmunity is the production of IFN-α. In the pathogenesis of SLE, type I IFN signals are considered to have an important role by inducing the expression of MHC II on antigen-presenting cells, expanding autoreactive T cells, and increasing the production of antibodies from B cells (31, 32). The increased expression of IFN-α genes by stimulation of the cGAS/STING pathway, which was not observed in Ly6C^lo^ cells, indicated that Ly6C^hi^ monocytes are a potential source of IFN-α. This is similar to the pristane-induced lupus model, in which the source of type I interferon is thought to be immature Ly6C^hi^ monocytes specifically induced by pristane administration (33). In humans, IFN-α was produced by monocytes *via* the cGAS/STING pathway, and its activation was correlated with disease activity in SLE (28). These findings suggest a role for Ly6C^hi^ monocytes in the induction of Tfh and autoantibody production *via* the stimulation of TLR7 and the cGAS/STING pathway. Therefore, increased Ly6C^hi^ monocyte-derived cells in lymph nodes in the induction phase through to the late phase might be involved in the development of autoimmunity.

Ly6C^lo^ monocytes, the mouse counterparts of human CD16^+^ non-classical monocytes, accumulate in inflamed kidneys during lupus nephritis and are thought to be pathogenic in kidneys (17). In a lupus model of ABIN1 (Tnip1)-deficient mice, which have a dysfunction in the regulation of NF-κB, nephritis occurred with the accumulation of Ly6C^lo^ monocytes in the kidneys. Tnip1^–/–^Rag1^–/–^ mice, which lack mature T cells and B cells, still developed glomerulonephritis, indicating Ly6C^lo^ monocytes are pathogenic and that adaptive immune system responses were not necessary for kidney pathology (4). In our study, Ly6C^lo^ monocyte-like cells were increased in long-term IMQ-treated kidneys and they originated, at least in part, from monocytes and appeared to have an inflammatory phenotype based on their upregulated inflammatory and chemokine genes including *Il6*, *Cxcl13*, and *Ccl5*.

Our monocyte transfer experiments demonstrated Ly6C^lo^ monocytes from IMQ-treated mice were more likely to infiltrate into organs, which might explain the increased number of Ly6C^lo^ monocyte-like cells in the kidneys of IMQ-treated mice. Gene expression analysis revealed peripheral Ly6C^lo^ monocytes in IMQ-treated mice had upregulated expressions of adhesion-related genes. Furthermore, the upregulation of *Ccl5* in the kidneys of Ly6C^lo^ monocyte-like cells was in accordance with the suggested involvement of the CCL5/CCR5 axis in the recruitment of Ly6C^lo^ monocytes in the CNS of another SLE model in FcγRIIB^-/-^ *Yaa* mice (25). Thus, IMQ-induced Ly6C^lo^ monocytes were likely to be primed to infiltrate into tissues and once they reached the kidneys, they had obtained an inflammatory and chemoattractive phenotype. Preventing monocytes from infiltrating the kidneys is a potential strategy for the treatment of nephritis although targeting chemokines and their receptors is still far from practical use (34, 35). Another possibility is targeting adhesion molecules such as integrins as Ly6C^lo^ monocytes that have upregulated *Itga9* and *Itgad*, as demonstrated in our study. Of note, the blockade of integrins might be a therapeutic target for rheumatoid arthritis and multiple sclerosis (36).

The roles of monocytes and MF in lupus nephritis require clarification. The classification of murine kidney mononuclear phagocytes, especially MF and DC, has long been confused, but recently CD64^+^ F4/80^+^ MHC II^+^ spindly processed cells in the interstitium were identified as MF (37), and resident MF were not replaced by bone marrow-derived cells in the steady state (21). Although resident MF were reported to be involved in the initiation of nephritis (38), it was not clear whether resident MF were involved in the long-term pathogenesis. In our study, short-term IMQ treatment caused the disappearance of MHC II^hi^ MF and the emergence of MHC II^lo^ F4/80^+^ cells, which did not accompany the influx of bone marrow-derived cells in the short-term. This was probably caused by the phenotypic change of resident MF in the kidneys, which may be related to the initiation of nephritis. In contrast, mono-like cells and MF in the kidneys were replaced by bone marrow-derived cells in the long-term. Ly6C^lo^ monocytes were more likely to infiltrate into the kidneys and differentiated to inflammatory mono-like cells or MF. In terms of the pathogenicity and fate of infiltrated monocytes, murine kidney CD11c^+^ myeloid cells, which were differentiated from Ly6C^lo^ monocytes, were reported to promote lupus nephritis by interacting with CD4^+^ T cells (39). A study of human lupus nephritis reported that CD11c^+^ myeloid cells in the urine and kidneys were induced by peripheral monocytes and were proinflammatory. Furthermore, these cells produced proinflammatory cytokines, including IL-6 and IL-1β, when stimulated by nucleic acid, and were recruited to the kidneys *via* the IP10/CXCR3 axis (24), in accordance with our study in which *Cxcr3* was upregulated in the monocytes of IMQ-treated mice. Another study of lupus nephritis by the single cell RNA-seq analysis of kidneys suggested infiltrating non-classical monocytes started as inflammatory blood monocytes and differentiated into phagocytes and then an alternatively activated phenotype (40). Our study supports this idea of a differentiation trajectory based on differences in the expressions of genes encoding cytokines, chemokines, and resident macrophage-specific genes among Ly6C^lo^ monocyte-like cells and MF in the kidneys of controls and IMQ-treated mice. Furthermore, MF in control and IMQ-treated mice appeared phenotypically different in terms of the expression of *Cxcl13*. The upregulation of *Cxcl13* in IMQ-treated mice was noteworthy because this was not observed in control mice. SLE patients with lupus nephritis were reported to have significantly higher levels of serum CXCL13 than controls (41). CXCL13 is also related to the formation of tertiary lymphoid tissues (42) and the local production of autoantibodies (43). Podocytes produce proinflammatory mediators upon stimulation through CXCL13, indicating it might be another pathogenic factor of lupus nephritis (44). This evidence underscores the pathogenic importance of monocyte-derived MF and their expression of *Cxcl13* in the development of lupus nephritis. In summary, resident MF initially responded to stimulation in the kidneys and were probably involved in the initiation of inflammation. Then gradually, Ly6C^lo^ monocytes infiltrated into the kidneys. These monocytes were likely to have more inflammatory features upon arrival, and would differentiate to less inflammatory MF although these monocyte-derived MF were different from resident MF and appeared to have inflammatory features on the basis of their expression of *Cxcl13*.

This study demonstrated the different temporal and spatial roles of Ly6C^hi^ and Ly6C^lo^ monocytes in an IMQ-induced lupus model. We found that Ly6C^hi^ cells were increased in the lymph nodes and upregulated IFN-α genes upon stimulation of the cGAS/STING pathway. Ly6C^lo^ cells were increased in the late phase, and were more likely to infiltrate into tissues and become inflammatory cells in the kidneys. These differences in the functions of monocytes in terms of disease phase and organ involvement should be taken into account when considering monocytes involved in the pathophysiology of SLE.

## Data Availability Statement 

The RNA-seq datasets presented in this study can be found in online repositories. The names of the repository/repositories and accession number can be found below The name of repository: DNA Data Bank of Japan (DDBJ) Accession number: DRA013503.

## Ethics Statement

The animal study was reviewed and approved by laboratory animal experimentation at Juntendo University School of Medicine.

## Author Contributions

SM and AN designed the experiments. AN, MM, and AA performed the experiments and analyzed the data. TK and GM especially contributed to the experiment of monocyte stimulation and analysis. AN drafted the manuscript. DN and AC critically revised the manuscript. All the authors approved and reviewed the final manuscript.

## Funding

Japan Society for the Promotion of Science (Grant-in-Aid for Scientific Research 19K24003 and 21H02964) and a grant from the Institute for Environmental & Gender-specific Medicine, Juntendo University. This research was supported by the Platform Project for Supporting Drug Discovery and Life Science Research [Basis for Supporting Innovative Drug Discovery and Life Science Research (BINDS)] from AMED under Grant Number JP17am0101102.

## Conflict of Interest

The authors declare that the research was conducted in the absence of any commercial or financial relationships that could be construed as a potential conflict of interest.

## Publisher’s Note

All claims expressed in this article are solely those of the authors and do not necessarily represent those of their affiliated organizations, or those of the publisher, the editors and the reviewers. Any product that may be evaluated in this article, or claim that may be made by its manufacturer, is not guaranteed or endorsed by the publisher.

## Supplementary Material

Supplementary Figure 1 Identification of monocytes and monocyte-like cells in the peripheral blood and kidneys. **(A)** Classification of cells in the peripheral blood. Cells in panel e are monocytes. Monocytes were further classified into Ly6C^hi^ monocytes and Ly6C^lo^ monocytes. **(B)** Most peripheral blood CD45^+^CD11b^+^Lin^-^ cells expressed CD115 and CX3CR1. **(C)** Classification of cells in the cervical lymph nodes. Lymph node monocyte-like cells were identified based on Lin^-^CD11b^+^ and the expressions of Ly6C and TLR7. **(D)** Classification of cells in the kidneys. CD11b^+^Lin^-^ cells were classified roughly into three subsets of cells according to the expressions of F4/80 and MHC class II. F4/80^+^ MHC II^hi^ cells and F4/80^-^MHC II^hi^ cells were considered macrophages and cDC, respectively. F4/80^-^MHC II^lo^ cells were identified as monocyte-like cells based on Lin^-^CD11b^+^ and the expressions of Ly6C and TLR7. **(A–D)** show representative plots of several experiments. mono-like: monocyte-like cells, MF, macrophages; cDC, conventional dendritic cells.

Supplementary Figure 2 Proteinuria in IMQ-induced lupus mice and CD11b expression in kidney monocyte-like cells and macrophages. **(A)** Nephritis was induced by the long-term application of IMQ, which was confirmed by proteinuria in C57BL/6 mice (left) and NZB/NZW mice (right). C57BL/6 mice were treated with 40 mg topical IMQ three times a week for 5 weeks. NZB/NZW mice were treated with 10 mg topical IMQ three times for 3 weeks. Symbols represent individuals and horizontal lines indicate the mean and SEM. *P < 0.05 by Student’s *t*-test. **(B)** Flow cytometry analysis of CD11b expression in Ly6C^lo^ monocyte-like cells and MF in the kidneys of control and IMQ-induced lupus mice. Representative histogram of fluorescence intensity in each subset is shown.

Supplementary Figure 3 Gating strategy to sort blood monocytes and kidney monocyte-like cells for RNA-seq analysis. Blood monocytes (Ly6C^hi^ and Ly6C^lo^) and kidney monocyte-like cells were sorted from control and IMQ-induced lupus mice. Lupus like-disease was induced by the application of 20 mg IMQ three times a week for 7 weeks. Representative plots of samples from control mice are shown.

Supplementary Figure 4 RNA-seq analysis of peripheral monocytes and kidney Ly6C^lo^ monocyte-like cells. Cluster analysis of differentially-expressed genes (DEG) classified genes into 20 clusters. B-Ly6C^hi^: blood Ly6C^hi^ monocytes, B-Ly6C^lo^: blood Ly6C^lo^ monocytes, K-Ly6C^lo^: kidney Ly6C^lo^ monocyte-like cells.

Supplementary Figure 5 Ly6C^lo^ monocytes and monocyte-like cells are increased in lupus NZB/NZW mice. **(A)** Ly6C^lo^ monocytes are increased in the blood of aged NZB/NZW mice. Monocyte-like cells also tended to be increased in the lymph nodes. **(B)** Ly6C^lo^ monocyte-like cells are increased in the kidneys of aged NZB/NZW mice. **(C)** Application of topical IMQ (10 mg, three times a week for 4 weeks) to NZB/NZW mice increased Ly6C^lo^ monocyte and monocyte-like cells in the peripheral blood and lymph nodes. **(D)** Application of topical IMQ increased Ly6C^lo^ monocyte-like cells in the kidneys of NZB/NZW mice. The numbers in the graph indicate the cell number in 100,000 live CD45^+^ cells. Symbols represent individuals and horizontal lines indicate the mean and SEM. *P < 0.05, **P < 0.01, and ***P < 0.01 by Student’s *t*-test.
