# Supplementary figures and images for: Different Spatial and Temporal Roles of Monocytes and Monocyte-Derived Cells in the Pathogenesis of an Imiquimod Induced Lupus Model
Source: Front Immunol. 2022 Mar 15;13:764557. doi: 10.3389/fimmu.2022.764557 (PMC8964788; doi:10.3389/fimmu.2022.764557)

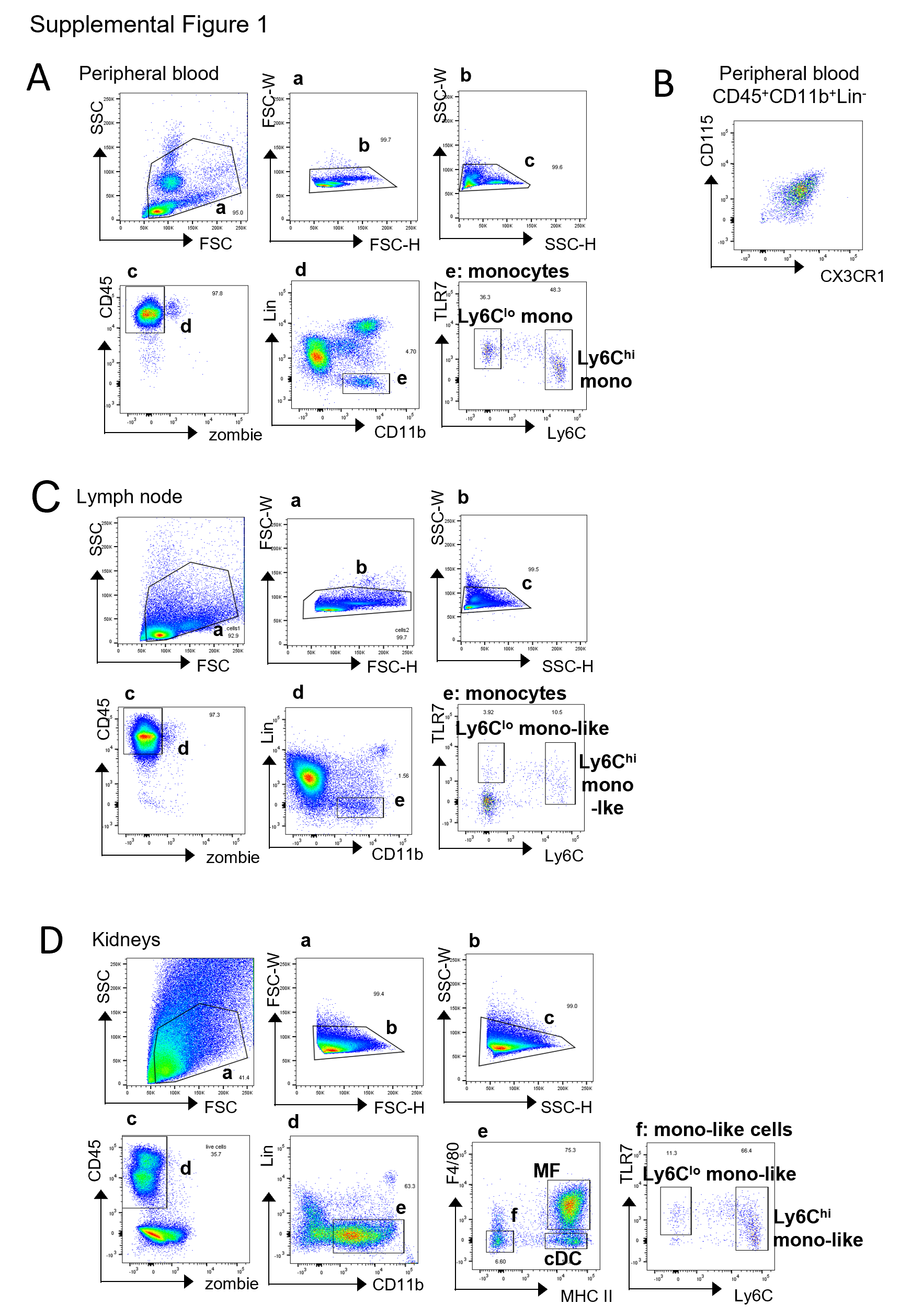

Supplement: Supplementary Figure 1 — Identification of monocytes and monocyte-like cells in the peripheral blood and kidneys. (A) Classification of cells in the peripheral blood. Cells in panel e are monocytes. Monocytes were further classified into Ly6Chi monocytes and Ly6Clo monocytes. (B) Most peripheral blood CD45+CD11b+Lin- cells expressed CD115 and CX3CR1. (C) Classification of cells in the cervical lymph nodes. Lymph node monocyte-like cells were identified based on Lin-CD11b+ and the expressions of Ly6C and TLR7. (D) Classification of cells in the kidneys. CD11b+Lin- cells were classified roughly into three subsets of cells according to the expressions of F4/80 and MHC class II. F4/80+ MHC IIhi cells and F4/80-MHC IIhi cells were considered macrophages and cDC, respectively. F4/80-MHC IIlo cells were identified as monocyte-like cells based on Lin-CD11b+ and the expressions of Ly6C and TLR7. (A–D) show representative plots of several experiments. mono-like: monocyte-like cells, MF, macrophages; cDC, conventional dendritic cells. [file Image_1.jpeg]

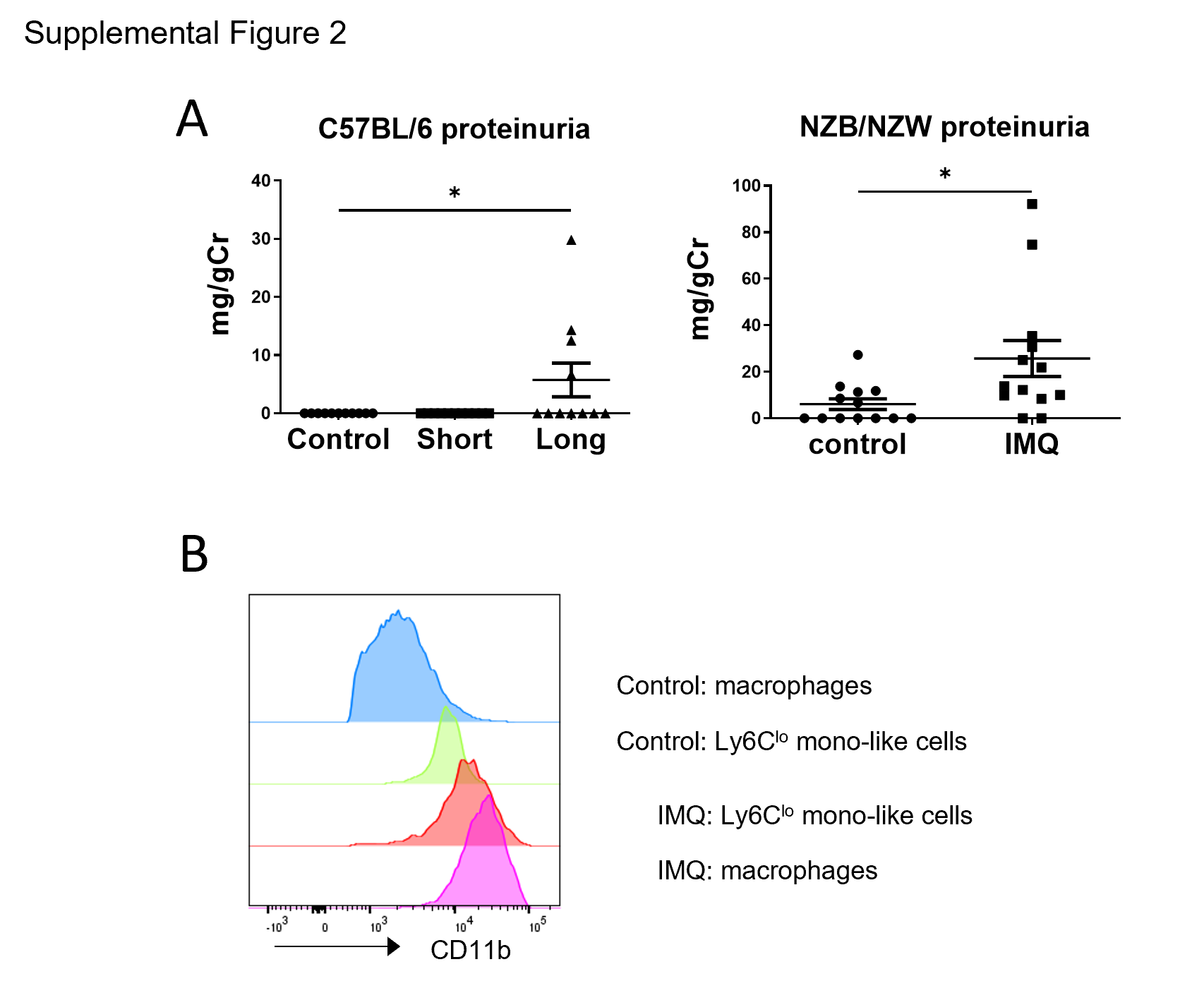

Supplement: Supplementary Figure 2 — Proteinuria in IMQ-induced lupus mice and CD11b expression in kidney monocyte-like cells and macrophages. (A) Nephritis was induced by the long-term application of IMQ, which was confirmed by proteinuria in C57BL/6 mice (left) and NZB/NZW mice (right). C57BL/6 mice were treated with 40 mg topical IMQ three times a week for 5 weeks. NZB/NZW mice were treated with 10 mg topical IMQ three times for 3 weeks. Symbols represent individuals and horizontal lines indicate the mean and SEM. *P < 0.05 by Student’s t-test. (B) Flow cytometry analysis of CD11b expression in Ly6Clo monocyte-like cells and MF in the kidneys of control and IMQ-induced lupus mice. Representative histogram of fluorescence intensity in each subset is shown. [file Image_2.jpeg]

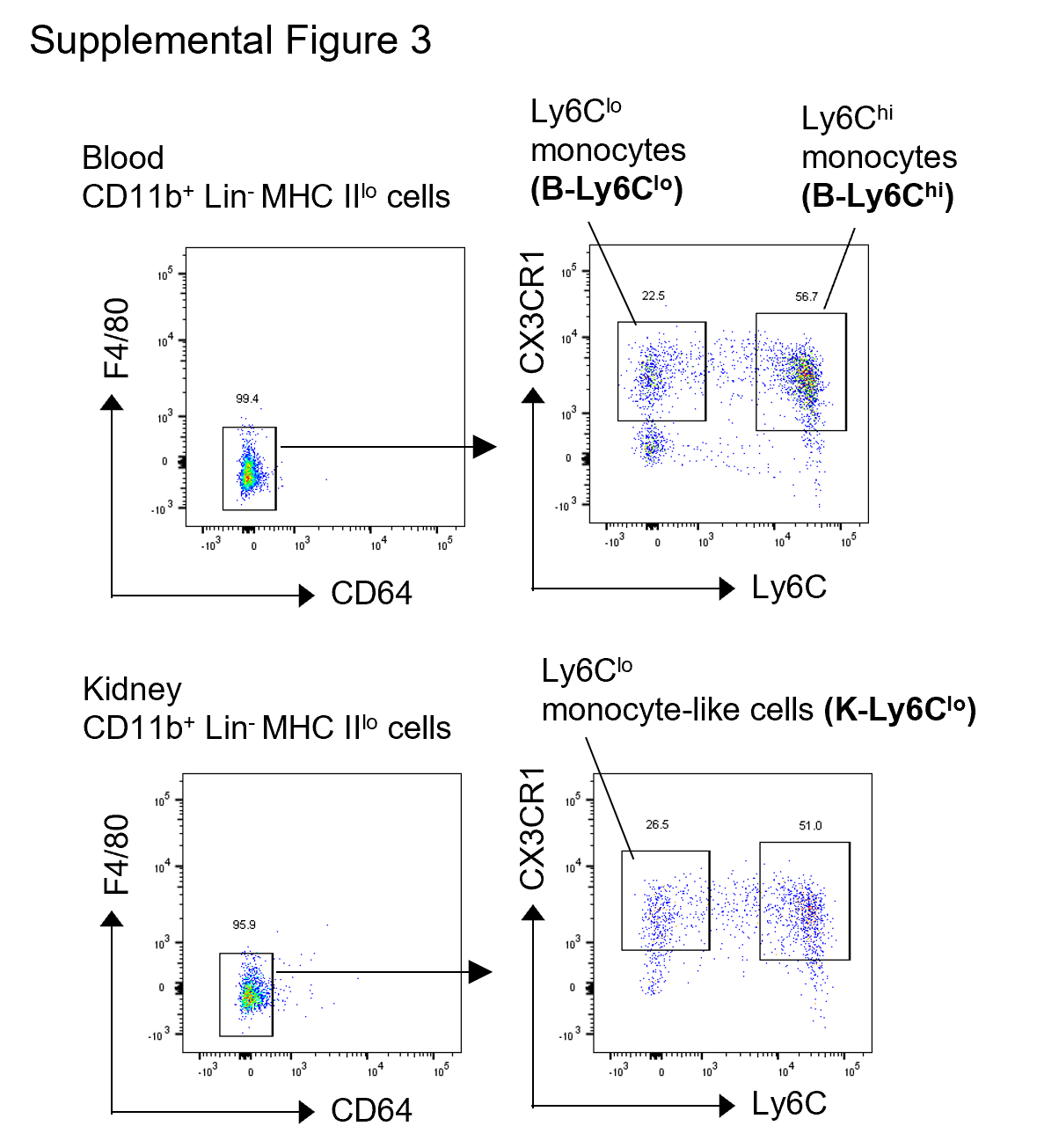

Supplement: Supplementary Figure 3 — Gating strategy to sort blood monocytes and kidney monocyte-like cells for RNA-seq analysis. Blood monocytes (Ly6Chi and Ly6Clo) and kidney monocyte-like cells were sorted from control and IMQ-induced lupus mice. Lupus like-disease was induced by the application of 20 mg IMQ three times a week for 7 weeks. Representative plots of samples from control mice are shown. [file Image_3.jpeg]

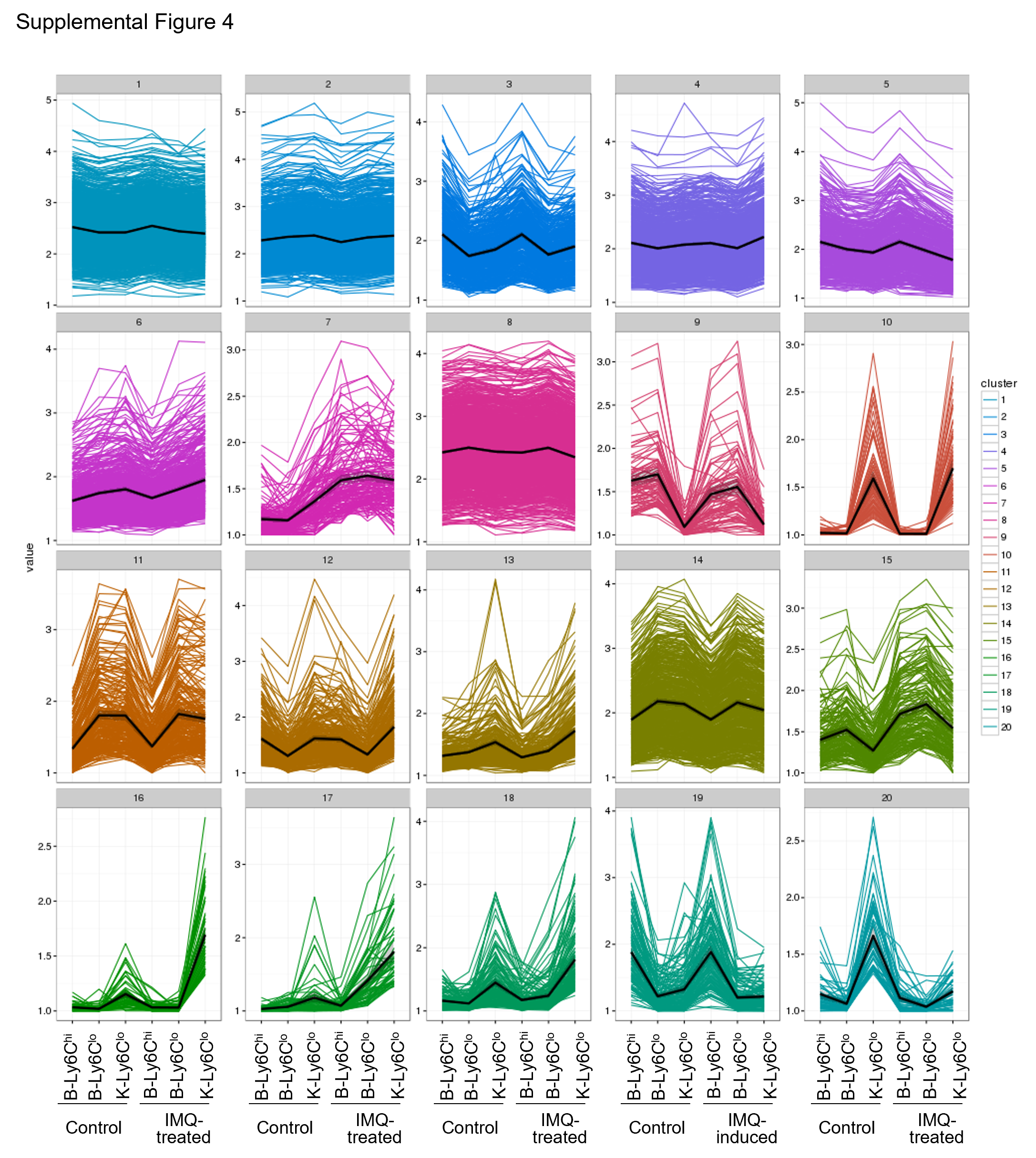

Supplement: Supplementary Figure 4 — RNA-seq analysis of peripheral monocytes and kidney Ly6Clo monocyte-like cells. Cluster analysis of differentially-expressed genes (DEG) classified genes into 20 clusters. B-Ly6Chi: blood Ly6Chi monocytes, B-Ly6Clo: blood Ly6Clo monocytes, K-Ly6Clo: kidney Ly6Clo monocyte-like cells. [file Image_4.jpeg]

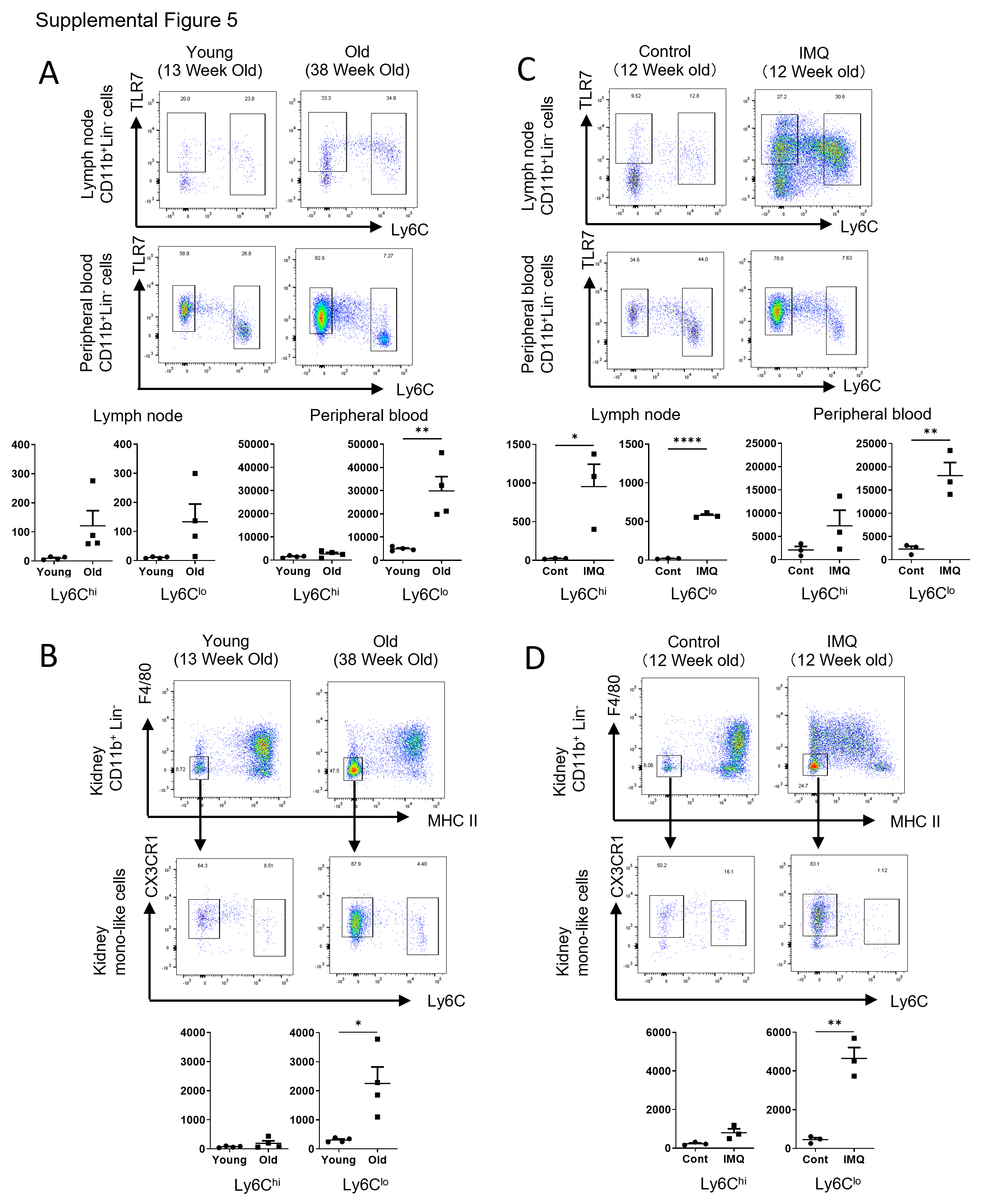

Supplement: Supplementary Figure 5 — Ly6Clo monocytes and monocyte-like cells are increased in lupus NZB/NZW mice. (A) Ly6Clo monocytes are increased in the blood of aged NZB/NZW mice. Monocyte-like cells also tended to be increased in the lymph nodes. (B) Ly6Clo monocyte-like cells are increased in the kidneys of aged NZB/NZW mice. (C) Application of topical IMQ (10 mg, three times a week for 4 weeks) to NZB/NZW mice increased Ly6Clo monocyte and monocyte-like cells in the peripheral blood and lymph nodes. (D) Application of topical IMQ increased Ly6Clo monocyte-like cells in the kidneys of NZB/NZW mice. The numbers in the graph indicate the cell number in 100,000 live CD45+ cells. Symbols represent individuals and horizontal lines indicate the mean and SEM. *P < 0.05, **P < 0.01, and ***P < 0.01 by Student’s t-test. [file Image_5.jpeg]
